# VALCOR: a protocol for the validation of SARS-corona virus-2 assays

**DOI:** 10.1186/s13690-022-00869-4

**Published:** 2022-03-29

**Authors:** Marc Arbyn, Sharonjit Kaur Dhillon, Marianna Martinelli, Cindy Simoens, Lize Cuypers, Jannes Bode, Marc Van Ranst, Philippe Corbisier, Jesper Bonde, Clementina Cocuzza

**Affiliations:** 1 Unit of Cancer Epidemiology/Belgian Cancer Centre, Scientific Institute of Public Health, Brussels, Belgium; 2 Department of Human Structure and Repair, Faculty of Medicine and Health Sciences, University of Ghent, Ghent, Belgium; 3 Laboratory of Clinical Microbiology and Virology, Department of Medicine and Surgery, University of Milano – Bicocca, Monza, Italy; 4 National Reference Centre for Respiratory Pathogens, Department of Laboratory Medicine, University Hospitals Leuven, Leuven, Belgium; 5 Laboratory of Clinical and Epidemiological Virology, Department of Microbiology, Immunology and Transplantation, Rega Institute for Medical Research, KU Leuven, Leuven, Belgium; 6 European Commission, Joint Research Centre, Directorate F – Health, Consumers and Reference Materials, Geel, Belgium; 7 Molecular Pathology Laboratory, Department of Pathology, Copenhagen University Hospital -Amager and Hvidovre Hospital, Copenhagen, Denmark

**Keywords:** SARS-CoV-2, Diagnostic test accuracy, COVID-19, Test validation, Quality control

## Abstract

**Background:**

Testing for SARS-CoV-2, together with vaccination, is one of the most vital strategies in curbing the current COVID-19 pandemic. The pandemic has led to an unprecedented need for diagnostic testing and the rapid emergence of an abundance of commercial assays on the market. Due to the nature of the pandemic and in the interest of health protection, many of these assays received provisional authorisation for emergency use without thorough validation. To limit false negative and false positive results, it is key to define common criteria that SARS-CoV-2 assays need to fulfil. VALCOR or “VALidation of SARS-CORona Virus-2 assays” is a protocol designed to set up a framework for test validation of SARS-CoV-2 virus assays.

**Objectives:**

VALCOR is a study protocol for the validation of assays used for confirmation of the presence of SARS-CoV-2 in patients with COVID-19 disease or the screening of carriers of SARS-CoV-2 virus by the identification of viral RNA in oropharyngeal and/or nasopharyngeal specimens or other specimens from the human respiratory tract.

**Methods:**

The VALCOR panel of samples will contain clinical human specimens and standardised artificial specimens. The collection of clinical specimens will include nasopharyngeal or oropharyngeal specimens or other specimens from the respiratory tract obtained from COVID-19 patients and healthy carriers of SARS-CoV-2 as well as specimens from subjects not carrying SARS-CoV-2. Artificial specimens include calibrated amounts of viral RNA of SARS-CoV-2 sequences provided by established competent agencies that produce reference materials for the assessment of the limit of detection of each assay. The panel of samples are sent from a central reference laboratory (having access to biobanks of clinical specimens tested already for SARS-CoV-2 with a reference comparator assay) to participating laboratories for testing with a SARS-CoV-2 index assay that requires evaluation.

**Discussion:**

VALCOR provides a harmonised and standard framework to benchmark the testing performance of SARS-CoV-2 assays that are rapidly evolving. As the pandemic incited an urgent need for testing capacity, there is a gap in the comprehensive validation of SARS-CoV-2 assays. This study will generate comprehensive validation data for assays used for the diagnosis of SARS-CoV-2 and may serve as a basis for other validation protocols.

**Supplementary Information:**

The online version contains supplementary material available at 10.1186/s13690-022-00869-4.

## Background

Coronavirus disease 2019 (COVID-19) is a potentially lethal respiratory disease, caused by the severe acute respiratory syndrome coronavirus 2 (SARS-CoV-2). COVID-19 was initially reported in Wuhan (Hubei province, China) where it caused a large scale, yet localized epidemic near the end of 2019 [1]. Since then, SARS-CoV-2 has spread rapidly across all continents and has evolved into a global pandemic, with more than 450 million confirmed cases and over six million deaths, as of March 2022 [2]. The World Health Organization (WHO) and other international and national health authorities worldwide have underlined the urgent need to increase testing capacity as a vital strategy to reduce transmission of SARS-CoV-2 [3].

Assays that detect infection with SARS-CoV-2 are generally divided into two categories. The first include nucleic acid amplification tests (NAATs). These types of tests are used to detect targeted sequences of the viral genome. For SARS-CoV-2, NAATs identify viral ribonucleic acid (RNA) sequences contained in respiratory samples mainly collected with nasopharyngeal or oropharyngeal swabs. The most common and widely used assay is the reverse transcription-polymerase chain reaction (RT-PCR), which combines reverse transcriptase of RNA into deoxyribonucleic acid (DNA) (referred to as complementary DNA or cDNA) followed by PCR amplification of specific DNA targets. Other amplification methods to detect SARS-CoV-2 include isothermal amplification such as transcription-mediated amplification (TMA) and loop-mediated isothermal amplification (LAMP). Whereas RT-PCR requires cycling at different temperatures, isothermal amplification occurs at a constant temperature. Most NAATs are laboratory-based and require handling by trained personnel. Some NAATs are rapid and can be performed at the point of care, outside specialized laboratory settings; these may however not be as accurate as laboratory-based NAATs. The second category of SARS-CoV-2 tests detect viral antigens, i.e. proteins that surrounds the viral genome. Like the NAATs, antigen tests are used to detect current infection of SARS-CoV-2. Antigen tests differ from NAATs as they are easier to perform and provide rapid results. However, antigen tests have a lower sensitivity when compared to NAATs. Currently, the “gold standard” for the diagnosis of SARS-CoV-2 remains the laboratory-based NAATs, specifically RT-PCR assays [4–6].

Several NAATs and antigen tests have been developed in response to the pandemic. Many health agencies have set up frameworks for rapid test validation and provisional authorization [7, 8]. Sufficient availability of validated tests is required not only for diagnosis and correct management of hospitalized COVID-19 patients but is crucial for the identification of infected individuals, contact tracing, surveillance of the spread of infection and may be pivotal in decision making regarding the estimation of population-based contagious levels. This in turn informs and guides authorities on non-pharmaceutical interventions (NPIs) such as school and workplace closures, stay at home orders, national and international travel restrictions as well as quarantine measures in place [9].

Given the urgency and need for large scale testing, many regulatory agencies have issued provisional authorisations applying validation criteria based on the limit of detection, non-comparative analytical performance without claims for target benchmarks, in silico genome sequence and cross-contamination. As many new assays have arrived on the market, health authorities require lists of well-validated assays and more comprehensive validation criteria are needed [10]. Additionally, with the prospect of SARS-CoV-2 circulating in multiple waves and concerns over new variants of concern, massive testing capacity will have to be allocated globally for years to come [11]. As the majority of assays currently on the market are approved by emergency measures if anything, we propose a structured, open-source approach to the validation of SARS-CoV-2 diagnostic assays, specifically NAATs. Hereinafter, the word “assay(s)” is used to refer to NAATs used for the detection of SARS-CoV-2 unless otherwise specified.

We therefore establish a validation framework for the systematic performance evaluation of SARS-CoV-2 assays. The VALCOR (acronym for VALidation of severe acute respiratory CORona virus 2 assays) protocol is inspired on VALGENT (VALidation of HPVGENotyping Tests) which is a successful forum for comparison and validation of human papillomavirus tests usable for cervical cancer screening [12, 13]. In this paper, we describe the VALCOR study protocol in detail.

## Methods

### Study design

This is a multi-centre cross-sectional diagnostic test accuracy study to assess the diagnostic parameters of SARS-CoV-2 assays for the detection of SARS-CoV-2. The VALCOR consortium entails a collaboration between Sciensano (the National Scientific Institute of Public Health and currently responsible for the coordination of the VALGENT network) in Belgium, together with virology laboratories (hereinafter referred to as provider laboratories), preferentially with the role of national reference centres for SARS-CoV-2 control in Europe. Provider laboratories will compile a VALCOR panel composed of clinical and artificial samples which will be tested with an established reference SARS-CoV-2 test at the provider laboratory. Well-defined aliquots prepared from the VALCOR samples will be sent to client laboratories for testing with an index SARS-CoV-2 test. Virological sensitivity and specificity of index tests to detect the presence of SARS-CoV-2 as defined by the reference test will be the main outcome. Limit of detection will be assessed on a series of dilutions of clinical and artificial specimens.

### Participating provider laboratories

Thus far, two VALCOR studies are in progress, a Belgian VALCOR and an Italian VALCOR. The Belgian VALCOR study has been approved by the Ethics Committee Research (EC Research) of University Hospitals Leuven (UZ Leuven) under the registration reference number S64233. The Italian VALCOR study has been approved by the Ethics Committee of the University of Milano – Bicocca under the registration reference number 0044362/20. Samples for the Belgian VALCOR are provided by the UZ Leuven, national reference laboratory for respiratory pathogens (Leuven, Belgium) and those for Italian VALCOR have been made available by the Clinical Microbiology and Virology Laboratory of the University of Milano-Bicocca (Monza, Italy).

### Study population

The protocol foresees including clinical samples and artificial samples. Clinical samples (nasopharyngeal, oropharyngeal or other specimens from the respiratory tract) are obtained from COVID-19 patients or healthy carriers of SARS-CoV-2 as well as subjects not carrying SARS-CoV-2. Artificial samples with calibrated amounts of viral RNA are included as external quality control for the analytical validation of assays. Details of included samples are described below;

### Composition of the VALCOR panel

A VALCOR panel will contain 220 clinical specimens (180 non-diluted and 40 diluted) as well as an additional number of dilutions of artificial standard reference viral RNA material.

1. Artificial specimens containing specified SARS-CoV-2 RNA sequences prepared by institutions specialised in the production of standard reference materials from microbiological agents (for instance Joint Research Centre of the European Commission [JRC] [14], National Institute of Standards and Technology [NIST] [15], American Type Culture Collection [ATCC] [16], BEI Resources [17] and others).
  A series of dilutions will be prepared from which the limits of detection and/or ranges of detectability will be assessed. The origin, definition and preparation of dilutions are described in the supplementary material (p2–4).
2. Clinical specimens: residual original rough material or extracted RNA after SARS-CoV-2 testing stored in biobanks of the VALCOR provider laboratories (reference laboratories for SARS-CoV-2 or other laboratories mandated by these reference laboratories). The panel of clinical specimens will be composed as described below;

- 40 samples derived from hospitalized, SARS-CoV-2 confirmed cases
- 50 samples derived from non-hospitalized, SARS-CoV-2 confirmed cases
- 90 samples derived from SARS-CoV-2 negative cases
- 40 diluted samples from 2b (4 dilutions [1:2, 1:10, 1:20,1:50] of 10 samples randomly selected from the 50 non-hospitalized patients).

Each fresh sample of the VALCOR panel will be divided into aliquots of the original material and stored at − 80 °C. Aliquots upon request will be sent to the client’s laboratories in order to validate their assays Data on the pre-analytical phase of the testing such as the sample retrieval process, transport and storage conditions and storage media type will be recorded in as much detail as possible as these steps can impact test performance.

### Testing procedures in VALCOR

One Belgian (VALCOR-BE1) and three Italian VALCOR panels have been compiled so far. The samples of VALCOR-BE1 were suspended in Universal Transport Medium (UTM) and Phosphate Buffered Saline (PBS). VALCOR-IT1 and VALCOR-IT2 were suspended in Universal Transport Medium (UTM) whereas the samples of VALCOR-IT3 were suspended in eNAT™ medium. Each sample of the VALCOR panels has to be tested with an established reference test in the provider laboratory and subsequently with index tests to be evaluated in client laboratories.

### Transport of material and transmission of data

#### Transport of specimens

**Fig. 1:**
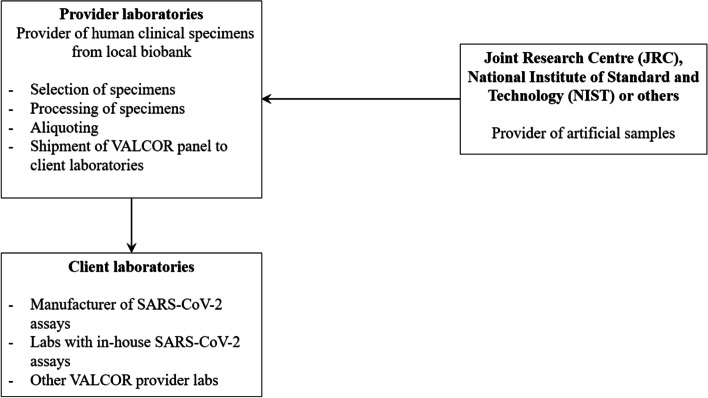
Overview of the flow of specimens within the VALCOR study

Transport of the VALCOR panel from the provider laboratory to the client laboratories will be regulated through the Material Transfer Agreement (Fig. 1).

#### Transmission of data

**Fig. 2:**
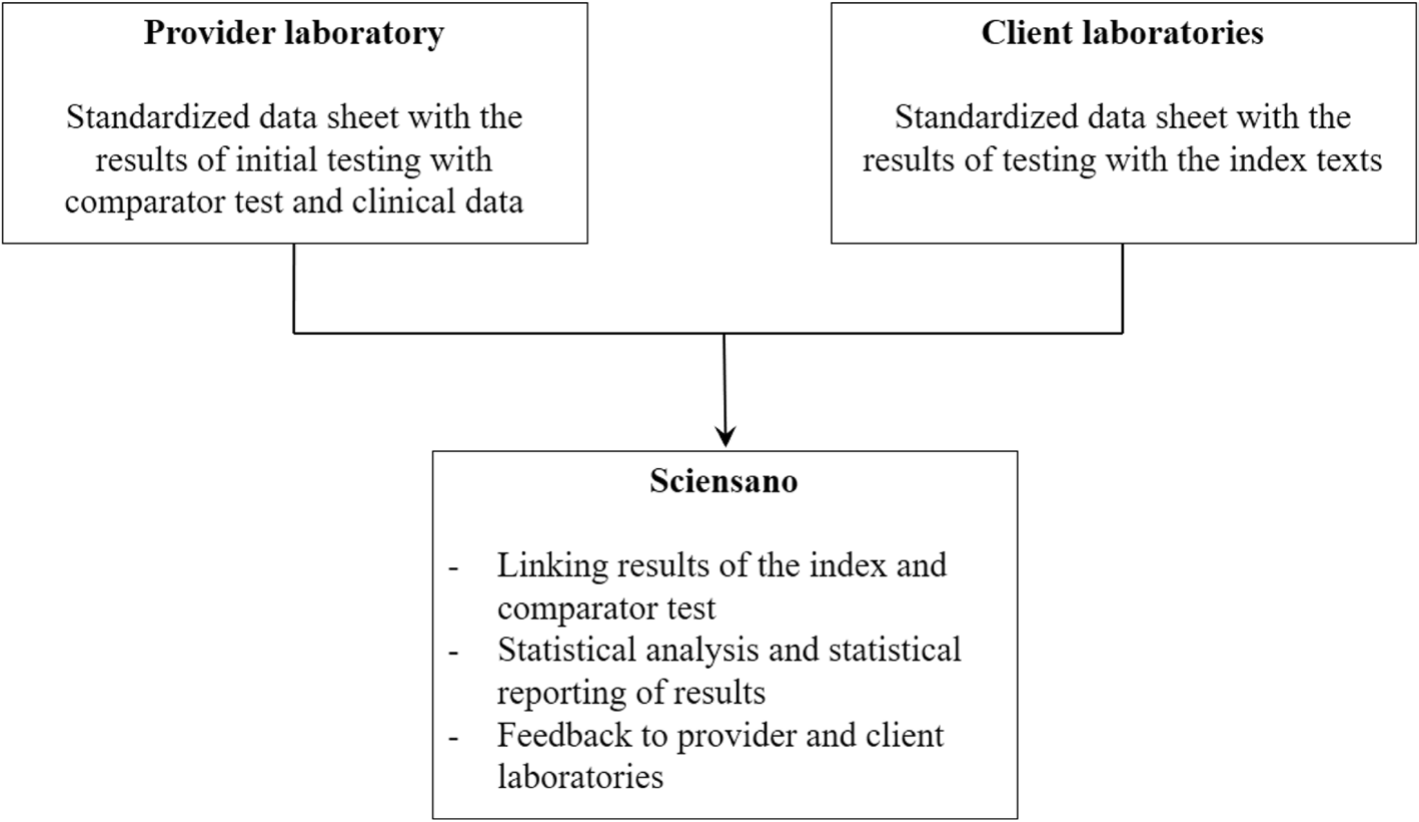
Overview of the flow of data within the VALCOR study

The provider laboratory will transmit data on clinical information and the results of the initial SARS-CoV-2 testing with the comparator assay described above in testing procedures to Sciensano (Fig. 2). The content of the data file is available in the supplementary material (p 5). The client laboratory will complete a standardised data sheet with the results of the SARS-CoV-2 testing with their assay and transmit this to Sciensano.

Standard data record sheets will be used by all VALCOR partners. Epidemiologists from the department of Public Health and Surveillance of the Sciensano institute (Brussels) will receive the virological results of index test(s) from each participating client laboratory and will link with the virological data of the reference test performed in the provider laboratory.

### Outcome measures and data analysis

The statistical analysis will subsequently be performed using Stata 16.0 (College Station, TX, USA).

Outcomes will be virological sensitivity and specificity of the index tests vs the comparator test. If output data from index and comparator tests include a quantitative metric (signal strength) related to viral load, scatterplots will be made which may be explored to assess possible cut-off optimisations.

Network meta-analyses using methods to pool multiple testing accuracy data will be applied as soon as results from different VALCOR panels will become available [18, 19]. The matrices of VALCOR test data will be enriched with data extracted from studies included in a systematic review and meta-analysis of published reports (peer-reviewed and grey literature).

After a few runs of VALCOR, one or more standard comparator tests might be chosen based upon consensus to be reached with the comity of collaborating VALCOR partners. Benchmark values for validation will be defined as well taking into account the use of the test (clinical diagnosis, tracking of contacts, screening in the population or defined communities, point-of-care testing). The focus will be on virological sensitivity.

## Discussion

The VALCOR protocol builds further on the experience of the VALGENT concept which is a successful forum for comparison and validation of human papillomavirus (HPV) tests usable for cervical cancer screening [12, 13]. The rationale and validation panel design principles of VALGENT is converted into a system to validate assays that detect SARS-CoV-2. VALCOR is the first validation study that will facilitate a standardised validation framework for systematic performance evaluation of SARS-CoV-2 assays.

Testing for COVID-19 is ultimately one of the key approaches to tackle and confine the SARS-CoV-2 pandemic. In particular, for both screening and contact tracing of communities, SARS-CoV-2 assays with a high sensitivity and negative predictive value (depending on the background prevalence) are needed. Specificity is important as well however good positive predictive values will be guaranteed in clinical settings given the clinical pre-triage and suggestive symptoms. Assay’s sensitivity and specificity also need to take into account the different sample types which are used for SARS-CoV-2 detection, such as nasopharyngeal, oropharyngeal, nasal swabs or less invasive saliva samples. Different suspension liquid or media (such as saline or PBS solution, UTM, viral transport medium (VTM), eNAT, etc.) may influence the stability of viral nucleic acid of the sample over time. Additionally, the pre-analytical procedures such as retrieval and transport, rapid or traditional nucleic acid extraction methods, the use of appropriate assays’ internal controls (to evaluate sample adequacy) may impact test performance [20, 21].

Since the emergence of variants of SARS-Cov-2, surveillance testing is now routinely carried out to monitor and track the emergence of new variants of concerns. As many countries now require positive samples to be further tested for whole-genome sequencing, there is a limited volume of residual material that is stored in reference laboratories. This may pose a limitation as there could be a lack of available clinical specimens required for validation studies such as VALCOR. Besides, specimens are usually stored in biobanks and retested. The stability of viral RNA over time could affect the assay’s results as degradation of virus material in frozen samples is expected.

As a result of the rapid spread of COVID-19, many regulatory bodies were forced to allow emergency authorisations for SARS-CoV-2 assays to fulfil the unprecedented need for testing requirements worldwide. The lack of vigorous validation procedures resulted in the subpar performance of many SARS-CoV-2 assays in the real-world setting. Our VALCOR protocol provides a basis for a standardised validation framework.

### Extensions of the VALCOR protocol

Nasopharyngeal samples are considered the reference specimen for the detection of SARS-CoV-2. However, alternate sampling methods such as saliva and oral swabs which are less invasive are a viable option for the diagnosis of SARS-CoV-2 as well. Hence, the validation of diagnostic tests using different samples is necessary to evaluate virological sensitivity and specificity of SARS-COV-2 assays on different types of specimens. VALCOR-like protocols may be adapted to include validation of other samples besides nasopharyngeal samples. Additionally, the VALCOR protocol may be adapted to accommodate other methods of testing besides NAAT such as antigen testing to detect SARS-CoV-2 proteins under the condition that viral proteins are conserved in the appropriate transport medium.

## Supplementary Information

**Additional file 1.**

## Data Availability

Final study datasets generated by VALCOR will be stored locally and securely at Sciensano. Anonymised will be made available by request to the corresponding author on a case-by-case basis pending approval from the information security coordinator at Sciensano.

## Abbreviations

COVID-19: Coronavirus disease 2019
SARS-CoV-2: Severe acute respiratory syndrome coronavirus 2
WHO: World Health Organization
NAATs: Nucleic acid amplification tests
RNA: Ribonucleic acid
RT-PCR: Reverse transcriptase-polymerase chain reaction
DNA: Deoxyribonucleic acid
TMA: Transcription mediated amplification
LAMP: Loop-mediated isothermal amplification
NPIs: Non-pharmaceutical interventions
VALCOR: Validation of severe acute respiratory coronavirus 2 assays
VALGENT: Validation of HPV genotyping tests
JRC: Joint Research Centre
NIST: National Institute of Standards and Technology
ATCC: (American Type Culture Collection)
LOD: Limit of detection
IVT: In vitro transcripted
ssRNA: Single-stranded ribonucleic acid
EQA: External Quality Control
RGTM: Research Grade Test Material
UTM: Universal Transport Medium
PBS: Phosphate Buffered Saline
HPV: Human Papilloma Virus
