# Supplementary material for: VALCOR: a protocol for the validation of SARS-corona virus-2 assays
Source: Arch Public Health. 2022 Mar 29;80:98. doi: 10.1186/s13690-022-00869-4 (PMC8962941; doi:10.1186/s13690-022-00869-4)
Supplement: Supplementary file 1 — Additional file 1. [file 13690_2022_869_MOESM1_ESM.pdf]

Contents

Information on artificial samples included in the VALCOR panel 2
Dilution protocol for the EURM-019 3
Dilution protocol for the NIST materials 3
Schematic diagram of the artificial samples 4
Standard datasheet used for the transmission of clinical and testing data 5
References 8

Supplementary appendix for

VALCOR: A protocol for the validation of SARS-corona virus-2 assays

Information on artificial samples included in the VALCOR panel

Dilutions of artificial RNA specimens to be used for determination of the limit of detection (LOD) on artificial samples.

1. EURM-019 is a solution containing a stabilised in vitro-transcripted (IVT) synthetic single-stranded RNA (ssRNA) of SARS-CoV-2 in buffer, prepared and developed by Joint Research Centre (JRC) for external quality control (EQA)(1). The synthetic RNA is 880 base pairs and contains sequences from the E, RdRP, N & S genes **(Supplementary Figure 1)** that can be transcribed and amplified by RT-PCR assays. The EURM-019 material is provided in vials containing approximately 100 µL of RNA solution at a concentration of about 7×10^7 copies/µL. This reference material needs to be diluted 700 times, in RNAse free water (Molecular Biology Grade), added with poly-A carrier RNA at 10 ng/µL, to achieve a dilution of 10^5 copies/µL. Four subsequent ten-fold serial dilutions can be prepared to achieve a total of 5 working solutions containing approximately: 10^5, 10^4, 10^3, 10^2 and 10 copies/µL to determine the LOD of the evaluated SARS-CoV-2 assays.

2. Research Grade Test Material ID: 10169 is composed of two unique synthetic RNA fragments from the SARS-CoV-2 genome (each ~4kb) in a background of 5 ng/µL human Jurkat RNA; they are provided in two separate tubes by NIST and include the SARS-Cov-2 sequences 25949-29698 and 12409-15962 from the USA-WA1/2020 isolate, respectively **(Supplementary Figure 2)(**2)**.** The NIST materials cover part of the ORF1ab, E and N target regions and may accommodate a larger number of commercial assays than the EURM-019 material. The concentration of each of the two synthetic RNA fragments, in the two separate tubes, is approximately 5×10^6 copies/µL in a total volume of 110 µL. Both vials of this reference material need to be initially diluted 1:50 in RNAse free water (Molecular Biology Grade), added with poly-A carrier RNA at 10 ng/µL, to achieve a dilution of 1×10^5 copies/µL. Four subsequent ten-fold serial dilutions can be prepared to achieve approximately: 10^5, 10^4 , 10^3, 10^2 and 10 copies/µL dilutions to determine the LOD of the evaluated SARS-CoV-2 assays.

Dilution protocol for the EURM-019

In particular, in order to prepare 2.5 ml volumes of each of the five serial dilutions (10^5, 10^4, 10^3, 10^2 and 10 copies/µL) starting from the original EURM-019 material proceed as follows:

- **1st step:** 10 µL of original EURM-019 material (7 x 10^7 copies/µL) + 690 µL RNAse free water with poly-A carrier RNA (1:70 dilution) = 10^6 copies/µL;
- **2nd step:** 250 µL of 10^6 copies/µL dilution + 2250 µL RNAse free water with poly-A carrier RNA = 10^5 copies/µL;
- **3rd step:** 250 µL of 10^5 copies/µL dilution + 2250 µL RNAse free water with poly-A carrier RNA = 10^4 copies/µL;
- **4th step:** 250 µL of 10^4 copies/µL dilution + 2250 µL RNAse free water with poly-A carrier RNA = 10^3 copies/µL;
- **5th step:** 250 µL of 10^3 copies/µL dilution + 2250 µL RNAse free water with poly-A carrier RNA = 10^2 copies/µL;
- **6th step:** 250 µL of 10^2 copies/µL dilution + 2250 µL RNAse free water with poly-A carrier RNA = 10^1 copies/µL

Dilution protocol for the NIST materials

In particular, in order to prepare 2.5 ml volumes for each of the five serial dilutions (10^5, 10^4, 10^3, 10^2 and 10 copies/µL) of both fragments of RGTM 10169 original material proceed as follows:

- **1st step:** 50 µL of original RGTM 10169 material (5 x 10^6 copies/µL) + 2450 µL RNAse free water with poly-A carrier RNA (1:50 dilution) = 1 x 10^5 copies/µL;
- **2nd step:** 250 µL of 105 copies/µL dilution + 2250 µL RNAse free water with poly-A carrier RNA = 10^4 copies/µL;
- **3rd step:** 250 µL of 104 copies/µL dilution + 2250 µL RNAse free water with poly-A carrier RNA = 10^3 copies/µL;
- **4th step**: 250 µL of 10^3 copies/µL dilution + 2250 µL RNAse free water with poly-A carrier RNA = 10^2 copies/µL;
- **5th step:** 250 µL of 10^2 copies/µL dilution + 2250 µL RNAse free water with poly-A carrier RNA = 10^1 copies/µL.

Schematic diagram of the artificial samples

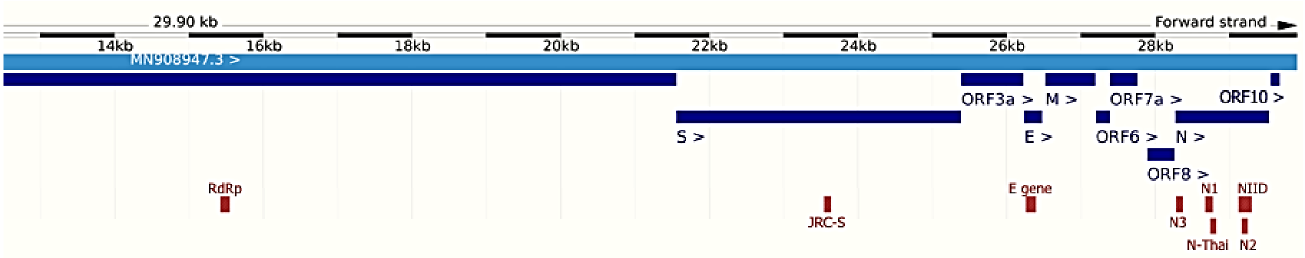

Supplementary Figure 1. Schematic of EURM-019 synthetic RNA fragments (Published with permission from JRC)

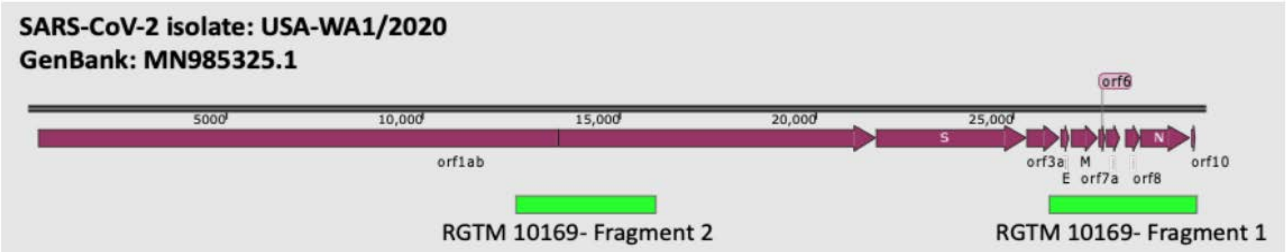

Supplementary Figure 2. Schematic of RTGM 101069 synthetic RNA fragments (Published with permission from NIST)

Standard datasheet used for the transmission of clinical and testing data

Datasheet_provider_v1

label	legend	
panel	VALCOR panel (ex. VALCOR_BE1, VALCOR_DK1, ...)	
num	Sample number (newly generated)	
mat	Material	
	1	extracted RNA
	2	rough residual material
extract	RNA extraction procedure used for test1	
anat	Anatomical site where the specimen was taken, or material	
	1	nasopharynx
	2	oropharynx
	3	oral cavity
	4	sputum
	5	saliva
	6	deep respiratory airway (trachea or deeper)
	9	unknown
dis	Disease status at moment of collection	
	1	very severe, needing admission at intensive care unit
	2	severe, needing hospitalisation
	3	moderate or mild disease, needing to stay isolated at home
	4	no symptoms
	9	unknown
place	Place or service where specimen collection took place	
	1	IC unit
	2	emergency unity of hospital
	3	preadminssion/triage centre for COVID-19 disease
	4	visit at cabinet of a doctor

	5	contact tracing
	6	elsewhere
	9	unknown
coldev	Device used to collect specimen	
	1	nasopharyngeal swab
	2	oropharyngeal swab
	3	lavage (fi for bronchoalveolar collection)
	5	oral swab
	8	vial to collect saliva
	9	unknown
col_d	Date of collection of specimen (format dd/mm/yyyy)	
age	Age of patient in years	
	999	unknown
sex	Sex of patient	
	1	male
	2	female
	3	other
	9	unknown
test1	first test used for identifying SARS-CoV-2	
genes1	Genes targeted for RNA SARS-CoV-2 testing	
load_x1	Viral load or signal strength output for test1 (to be repeated for each gene)	

Datasheet_client

label	legend
panel	VALCOR panel (ex. VALCOR_BE1, VALCOR_DK1, ...)
num	Sample number (newly generated by provider)
receive_d	Date of reception of VALCOR panel at client lab
	format: dd/mm/yyyy
mat	Material
	1 extracted RNA
	2 rough residual material
extract	RNA extraction procedure used for test, if client received rough residual material
test	Test used by client laboratory for identifying SARS-CoV-2 (to be detailed in form Test characteristics)
result	Result
	0 negative
	1 positive
	8 invalid
	9 unknown
genes	Genes targeted for RNA SARS-CoV-2 testing
load_x	Viral load or signal strength output for test1
	(to be repeated for each gene)
test_d	Date of testing of VALCOR panel at client lab
	format: dd/mm/yyyy

References

1. Joint Research Centre (JRC). EURM-019 single stranded RNA (ssRNA) fragments of SARS-CoV-2 [Available from: https://crm.jrc.ec.europa.eu/p/EURM-019 Accessed 10 August 2020.

2. National Institute of Standards and Technology (NIST). SARS-CoV-2 Research Grade Test Material [Available from: https://www.nist.gov/programs-projects/sars-cov-2-research-grade-test-material Accessed 10 August 2020.
